# South Asian Aging Brain Initiative: Neuroimaging

**DOI:** 10.1002/alz.091940

**Published:** 2025-01-09

**Authors:** Auriel A. Willette, Karthik Kota, William T. Hu

**Affiliations:** ^1^ Rutgers‐Robert Wood Johnson Medical School, New Brunswick, NJ USA; ^2^ Rutgers Institute for Health, Health Care Policy, and Aging Research, New Brunswick, NJ USA

## Abstract

**Background:**

South Asian (SA) older adults are one of the fastest growing US populations developing Alzheimer’s disease (AD) and related dementias (ADRD). Compared to non‐Hispanic white (NHW) Americans, SA are hesitant to enroll in neuropsychological and MRI research. This status complicates accurate assessment and diagnosis. Yet, SA may show differences in neurodegeneration patterns that elucidate novel brain‐behavioral relationships or network dysfunctions in multilingual speakers.

**Method:**

As part of the South Asian Aging Brain (SAAB) study, we recruited 62 non‐demented SA participants without subjective cognitive concerns to undergo English‐based neuropsychological testing and brain MRI. Freesurfer 7.4.1 was used to derive bilateral volume or cortical thickness in cortical and subcortical regions. After calculating Z‐scores based on National Alzheimer’s Coordinating Center (NAAC) calculators, stepwise linear regression was used to examine adjusted SAAB z‐scores with imaging outcomes. Covariates included age, sex, years of education, age at immigration, and years spent in the United States.

**Result:**

Among 29 women and 33 men, mean age was 64.9 years (range 24‐86y) with an average of 17.6 years of education (range 12‐20y) and US residency lasting 32.6 years (range 4‐59y). For Factor 1 (Semantic Memory), superior parietal gyrus, inferior parietal gyrus, and precentral gyrus loaded (adjusted R^2^=0.261; p<.01). For Factor 2 (Word Recall), only the bank of the superior temporal sulcus loaded (adjusted R^2^=0.231, p<.01). For Factor 3 (Story Recall), several areas explained recall including entorhinal cortex, precuneus, caudal anterior cingulate gyrus, medial orbital gyrus, and others (adjusted R^2^=0.640, p<.001). For Factor 4 (Attention), the inferior and superior parietal lobules loaded (R^2^=0.207, p<.01). For Factor 5 (Executive Function), postcentral gyrus and the inferior lateral ventricle loaded (adjusted R^2^=0.142, p<.05). Finally, for Factor 6 (Letter Fluency), areas included inferior parietal gyrus, lateral occipital gyrus, and bilateral cerebellar white matter (adjusted R^2^=0.311, p<.001).

**Conclusion:**

The results suggest that word and especially story recall are substantially explained by regions implicated in AD, such as frontal, temporal, and parietal areas. Tests associated with frontal brain regions (executive function, fluency) in NHW adults are more associated with parietal volume or cortical thickness in SA adults. Future work will need to explore this difference.

